# Rotator cuff repair using a bioresorbable nanofiber interposition scaffold: a biomechanical and histologic analysis in sheep

**DOI:** 10.1016/j.jse.2021.07.018

**Published:** 2021-08-25

**Authors:** Anthony Romeo, Jeremiah Easley, Dan Regan, Eileen Hackett, James Johnson, Jed Johnson, Christian Puttlitz, Kirk McGilvray

**Affiliations:** a Rush University Medical Center, Chicago, IL, USA; b Preclinical Surgical Research Laboratory, Colorado State University, Fort Collins, CO, USA; c Orthopaedic Bioengineering Research Laboratory, Colorado State University, Fort Collins, CO, USA; d Nanofiber Solutions, Columbus, OH, USA

**Keywords:** Rotator cuff repair (RCR), scaffold, nanofiber, ovine model, tendon, acute model

## Abstract

**Background:**

The purpose of this study was to evaluate the mechanical, structural, and histologic quality of rotator cuff repairs augmented with an interposition electrospun nanofiber scaffold composed of polyglycolic acid (PGA) and poly-L-lactide-co-ε-caprolactone (PLCL) in an acute sheep model.

**Methods:**

Forty acute infraspinatus tendon detachment and repair procedures were performed in a sheep infraspinatus model using a double-row transosseous-equivalent anchor technique either with an interposition nanofiber scaffold composed of polyglycolic acid–poly-L-lactide-co-ε-caprolactone or with no scaffold. Animals were euthanized at the 6-week (20 samples) and 12-week (20 samples) postoperative time points to assess the biomechanical and histologic properties of the repairs and to compare differences within each group.

**Results:**

Within the scaffold-treated group, there was a significant increase in ultimate failure force (in newtons) from 6 to 12 weeks (*P* < .01), a significant increase in ultimate failure load from 6 to 12 weeks (*P* < .01), and a significant increase in ultimate failure stress (in megapascals) from 6 to 12 weeks (*P* < .01). At 6 weeks, the tendon-bone attachment was most consistent with an “indirect” type of insertion, whereas at 12 weeks, a visible difference in the progression and re-formation of the enthesis was observed. Compared with controls, animals in the scaffold-treated group displayed an insertion of the fibrous tendon with the humeral footprint that was beginning to be organized in a manner similar to the “native” direct/fibrocartilaginous insertion of the ovine infraspinatus tendon. In the majority of these animals treated with the scaffold, prominent perforating collagen fibers, similar to Sharpey fibers, were present and extending through a region of calcified fibrocartilage and attaching to the humeral footprint. No surgical complications occurred in any of the 40 sheep, including delayed wound healing or infection.

**Conclusions:**

In a sheep acute rotator cuff repair model, securing a nanofiber scaffold between the tendon and the bone using a double-row transosseous-equivalent anchor fixation technique resulted in greater failure strength. Additionally, at the enthesis, Sharpey fiber–like attachments (ie, collagen fibers extending from the tendon into the calcified fibrocartilage of the humerus) were observed, which were not seen in the control group.

**Level of evidence:**

Basic Science Study; Biomechanical/Histology

Acute and chronic rotator cuff tears, both partial and complete, affect a significant number of patients each year. Surgery to treat a torn rotator cuff tendon is the most common surgical procedure related to the shoulder, with a current estimate of 500,000 cases annually in the United States, increasing to >600,000 cases by 2025.^32^ Techniques to augment surgical tendon repairs of the rotator cuff have primarily focused on improving the mechanical attachment at the tendon-bone interface for decades. For example, increasing the tensile strength of the sutures^17^ and modifying the surgical technique to create stronger and/or larger bone-tendon contact^9^ have previously been performed.

Although the clinical results following rotator cuff repair are good, a consistent issue with surgical treatment of rotator cuff tears is the variable healing rate after surgical repair.^5^ Furthermore, studies have consistently shown improved functional outcomes when the surgical repair recapitulates the native structure of the healthy tendon compared with when it does not.^14,43^ Because of the inconsistent healing despite significant improvements in mechanical fixation, biological strategies to augment the surgical repair, including bioresorbable scaffolds, have been introduced in an effort to improve the rate of tendon healing.^39^

Scaffolds are bioabsorbable materials that function as a temporary structure that acts to preserve physiological and/or biologically active molecules owing to its architecture and physicochemical properties encouraging restoration or regeneration of native tissues. Recently, the electrospinning of bioabsorbable synthetic polymers has allowed the creation of microporous structures with fiber diameters within the nanometer range (referred to as “nanofiber scaffolds”). These nanofiber scaffolds have been shown to encourage the development of normal tendon-bone enthesis architectures demonstrated through their ability to support cellular migration, infiltration, and proliferation.^47,59^ The augmented healing response is further supported by the gradual degradation of the scaffold such that it can be replaced with new tissue as the healing process proceeds toward completion.^2,20^

Therefore, the purpose of this study was to report the biomechanical and histologic outcomes following a standardized double-row transosseous-equivalent rotator cuff repair in a sheep model of acute rotator cuff tear using a novel nanofiber scaffold as an interposition scaffold at the site of tendon-bone healing. We hypothesized that this nanofiber scaffold composed of a microporous, nonwoven, bioabsorbable biphasic polyglycolic acid (PGA) and poly-L-lactide-co-ε-caprolactone (PLCL) polymer would encourage a healing environment that would allow for collagen fiber integration into bone without scar interposition, resulting in improved structural and mechanical properties of the tendon-bone interface. Furthermore, with the formation of Sharpey fibers for collagen-bone integration, the biomechanical strength would likely be improved at earlier stages in the healing process and surpass the biomechanical strength in the control group.

## Materials and methods

**Figure 1:**
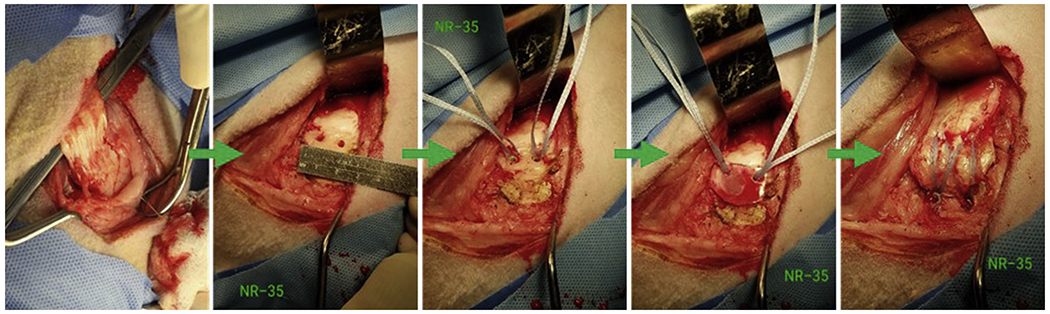
Representative images demonstrating modified double-row technique in which the sharply transected infraspinatus tendon (*left*) was immediately reattached using a total of 4 suture anchors (*right*) in an acute transection and reattachment ovine model. This sequence of images also shows the placement of the scaffold at the enthesis (*fourth panel*) prior to tendon reattachment.

The acute ovine infraspinatus model has previously been demonstrated to be a reliable translational research platform for the human supraspinatus tendon.^8,15,22,23,37,38,40–42,49,50^ Forty skeletally mature (age, 3-5 years; weight, 65-115 kg) female Columbia Cross sheep (*Ovis aries*) underwent rotator cuff repair using an open approach. Florfenicol (20 mg/kg subcutaneously), phenylbutazone (1 g orally), and 2 fentanyl patches (100 and 50 μg transdermally) were applied to all sheep 24 hours prior to surgery and maintained for 5 days. The auricular vein and artery were catheterized, and anesthesia was induced with a combination of ketamine (3.3 mg/kg intravenously) and diazepam (0.1 mg/kg intravenously). Following anesthetic induction, the sheep were intubated with a cuffed endotracheal tube, placed in right lateral recumbency, and maintained on isoflurane (1.5%-3%) with 100% oxygen using positive pressure ventilation (20 cm H_2_O) for the duration of the procedure. The animals were placed under general anesthesia. By use of the modified double-row technique,^25^ the humeral footprint was prepared to remove any remaining soft tissue, creating a bleeding bone bed. The sharply transected infraspinatus tendon was immediately reattached using a total of four 4.75-mm suture anchors (SwiveLock; Arthrex, Naples, FL, USA) as previously described^8,26,29^ (Fig. 1). The animals were randomly assigned to 1 of 2 treatment groups: (1) augmentation with a nanofiber scaffold device (Rotium; Nanofiber Solutions, Columbus, OH, USA) placed between the tendon and the bone (ie, an inlay position) and (2) no augmentation device (no scaffold). In the nanofiber group, the suture tape from the medial anchors was passed through the scaffold and then through the undersurface of the tendon at the corresponding position for the medial footprint. Next, 1 limb of suture tape from each anchor was passed through another anchor, which was fixed laterally on the footprint over the top of the tendon, compressing the tendon over the scaffold to hold both the scaffold and tendon with secure fixation. If the scaffold extended laterally to the repaired tendon, the excess scaffold was excised. Following recovery from the surgical procedure, the sheep could move and eat ad libitum for the entirety of the study period.

Twenty animals were euthanized at either 6 weeks or 12 weeks postoperatively to assess the temporal healing response (10 animals per treatment group per time point). At euthanasia, the infraspinatus muscle–scapula–humerus construct was isolated and harvested. A total of 10 contralateral untreated shoulders were also opportunistically collected to serve as “time zero” baseline biomechanical and histologic samples. Harvested humerus-infraspinatus constructs underwent dissection to remove any extraneous soft tissues, isolating the infraspinatus tendon from the associated muscle belly and skeletonizing the humerus. Repair sutures and the tendon-bone enthesis were left intact following the dissection.

We allocated 10 samples for biomechanical testing (5 in nanofiber group and 5 in no-scaffold group) and 10 samples for histologic analysis (5 in nanofiber group and 5 in no-scaffold group) from each time point. The 10 contralateral samples were equally allocated to either biomechanical or histologic study. The researchers performing biomechanical and histologic testing were blinded to the treatment groups and sacrifice time points until final statistical analyses were performed.

### Scaffold fabrication

**Figure 2:**
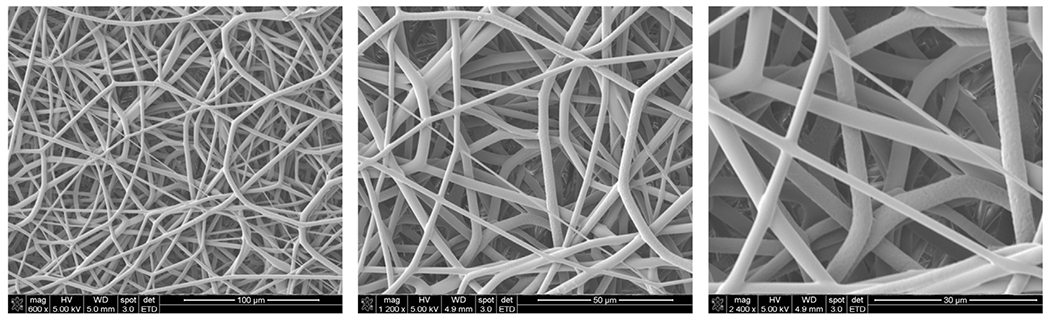
Representative scanning electron microscope images demonstrating architecture of nanofiber scaffold used in study. The scaffold is a nonwoven, biphasic, microporous nanofiber matrix composed of biodegradable polymer fibers consisting of polyglycolic acid and poly-L-lactide-co-ε-caprolactone.

The nanofiber scaffold (Rotium) used in this study is a nonwoven, microporous nanofiber matrix composed of biodegradable polymer fibers consisting of PGA and PLCL (width × length × thickness, 20 mm × 20 mm × 0.6 mm) (Fig. 2). Rotium is a Food and Drug Administration–cleared medical device that has passed all parts of the ISO-10993 biocompatibility panels. Succinctly, PGA was chosen to degrade within the first several days of implantation, which corresponds to the rate at which cells can infiltrate within the scaffold. Secondly, glycolic acid is a known modulator of macrophages and stimulates a pro-healing response instead of a fibrotic scar response.^3^ PLCL has a longer resorption time frame to help facilitate a gradual transition of mechanical forces from the scaffold to the newly deposited tissue. Additionally, lactic acid is known to have antioxidant and angiogenic properties.^21^ The combination of these 2 polymers provides a potent regenerative scaffold in vivo. The final appearance of the unwoven matrix mimics collagen-based extracellular matrix.^55^ Similar scaffold constructs have been demonstrated with in vitro cell cultures and with other animal models to support accelerated cellular migration, cellular infiltration, and facilitation of functional tissue regeneration.^57^ The 2 components of the nanofiber scaffold, PGA and PLCL, have differential degradation rates, creating a biphasic material that is replaced with neo-native tissue within 3-4 months.^57^ The highly porous and flexible initial scaffold provides a robust material that can tolerate surgical manipulation while maintaining the biocompatibility that allows for cellular adhesion, migration, and infiltration. After the initial 2-4 weeks following implantation, the scaffold becomes increasingly porous, facilitating a more advanced healing response including the continued viability of cellular infiltration with the initiation of native fiber formation through the boundaries of the scaffold.^57^

### Destructive biomechanical testing

The cross-sectional area (in square millimeters) of each tendon was calculated using an area micrometer, and the geometric mean was calculated for the tendon region of interest.^7,8^ The humerus of each sample was potted and mounted into a servo-hydraulic testing machine (MiniBionix 858; MTS Systems, Eden Prairie, MN, USA) using a specially designed cryo-clamp for biomechanical testing.^7,8,38^ Once mounted in the testing system, samples were manually tensioned to 10 N and the distance from the humerus enthesis to the cryo-clamp was recorded as the initial gauge length (in millimeters). Samples were then preconditioned in uniaxial tension (10 cycles between 0% and 2% strain) to minimize viscoelastic effects. Following preconditioning, a quasistatic displacement (100 mm/min) was applied to the tendon, aligned with the physiological loading direction, until sample failure. Force (in newtons) and displacement (in millimeters) data were collected at 100 Hz. Material properties (elastic modulus [in megapascals] and failure stress [in megapascals]) were calculated from structural measurements (ie, failure load [in newtons] and construct stiffness [in newtons per millimeter]) by normalization to cross-sectional area (in square millimeters) or to the initial gauge length (in millimeters) of the sample.

### Histologic analysis

For the histologic samples, the humerus-infraspinatus constructs were fixed (10% neutral buffered formalin for ≥7 days), dehydrated in graded solutions of ethanol (Tissue-Tek VIP; Sakura, Torrance, CA, USA), and then cleared with acetone and polymerized into a hardened plastic block (Hard Acrylosin; Dorn and Hart Microedge, Villa Park, IL, USA). By use of standard cutting and grinding techniques, 3 sections, separated by 500-800 μm, were created in the sagittal plane to display the humeral bone, repair site (ie, enthesis), implant (if applicable), and surrounding tendon soft tissue. Sections were stained with Sanderson rapid bone stain (which provides differentiation of cells within the section and allows detection of cartilage) and then counterstained with Van Gieson bone stain (allowing the differentiation of collagen and detection of bone [immature woven bone and mature lamellar bone]).^6^ Digital images of each section were acquired by field (Nikon E800 microscope [AG Heinze, Lake Forest, CA, USA] and Spot digital camera [Diagnostic Instruments, Sterling Heights, MI, USA]).

A standardized histomorphometric area was set within the surgical region of interest. Slides were semiquantitatively scored by a blinded, individual board-certified veterinary pathologist, using a modified ISO-1099/6 scoring rubric to assess biocompatibility, including cellular response and inflammation and/or foreign body response, as well as implant degradation. Additionally, a scoring system was devised, based on an adaptation of parameters from the Movin and Bonar scoring systems, for specific assessment of tendon histologic characteristics, including collagen fiber structure, tenocyte reactivity, stromal cell proliferation, and neo-vascularization.^7,8^

### Statistical analysis

A power analysis was performed using biomechanical data (ie, stiffness, in newtons per millimeter) from an analogous study examining acute rotator cuff tendon repairs augmented with an interposition bioresorbable scaffold with a vented anchor in the same ovine model.^8^ A 1-way analysis-of-variance power calculation indicated that a sample size of n = 5 (with delta = 84.38, standard deviation = 34.45, and power = 0.8) was needed per group to reach statistical fidelity of post hoc analysis (Minitab, version 18.1; Minitab, Chicago, IL, USA).

A standard 2-way analysis of variance was performed to determine statistical significance (SigmaPlot, version 11.0; Systat Software, San Jose, CA, USA) for all outcome parameters, with time and treatment as the independent variables. *P* < .05 was considered the level of significance. Untreated data were not included in the statistical analyses; however, the data are presented for baseline comparative purposes.

## Results

All 40 sheep were included in the final analysis. No evidence of infection or incision dehiscence was noted during the entire study period in any sheep. No lameness was observed following a 2-week recovery period. No grossly abnormal pathologies or abnormal tissue reactions were noted at the time of dissection, and no experimental issues were noted. All biomechanical tests were run to completion in all 40 animals (100.0%). Data figures are displayed in box-and-whisker plot format. The box is defined by the first and third quartiles. The whisker demarcation represents the maximum and minimum values.

### Biomechanical results

**Figure 3:**
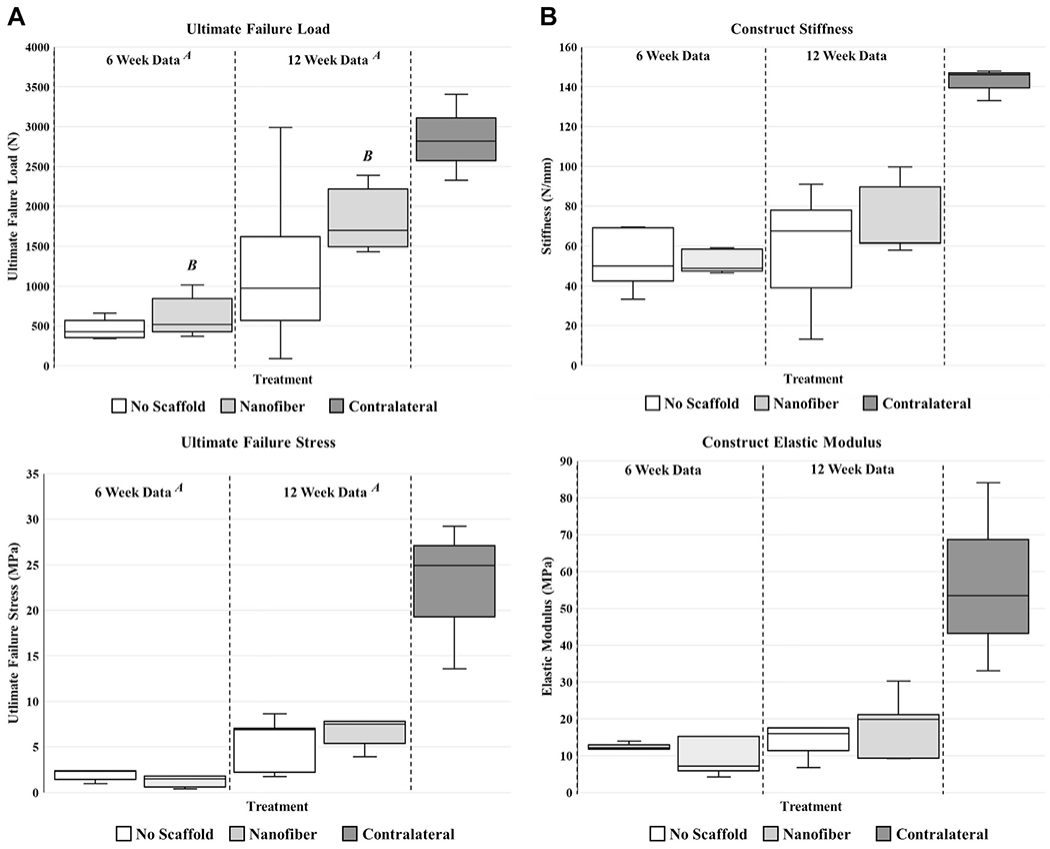
Ultimate failure load (*top left*), construct stiffness (*top right*), ultimate failure stress (*bottom left*), and construct elastic modulus (*bottom right*). There was a significant increase in ultimate failure load (in newtons) from 6 to 12 weeks (**A**, *P* < .01). Additionally, there was a statistically significant increase in only the nanofiber treatment group between 6 and 12 weeks (**B**, *P* < .01). There were no significant differences in construct stiffness (in newtons per millimeter), allowing for the effects of time (*P* = .41), treatment (*P* = .16), or the interaction between treatment and time (*P* = .36). There was a significant increase in ultimate failure stress (in megapascals) from 6 to 12 weeks (**A**, *P* < .01). There was no significant difference in construct elastic modulus (in megapascals), allowing for the effects of time (*P* = .91), treatment (*P* = .18), or the interaction between treatment and time (*P* = .89).

The data from the contralateral samples were not included in the statistical analysis; however, the data were displayed in the figures to have a better understanding of the healthy (“normal”) tissue biomechanical response (Fig. 3). A statistically significant increase in ultimate failure force (in newtons) was observed from the 0- to 6-week time point (*P* < .01), indicating a general increase in failure load magnitude with time (Fig. 3). At the 6-week point, the mean ultimate failure force was 634.51 ± 280.92 N for the nanofiber treatment vs. 470.49 ± 141.31 N for the control (no scaffold). Additionally, there was a statistically significant increase within the nanofiber treatment group between 6 and 12 weeks (*P* < .01), demonstrating a notable increase in ultimate failure load with this treatment (Fig. 3). At the 12-week point, the mean ultimate failure force was 1846.77 ± 431.96 N for the nanofiber treatment vs. 1247.00 ± 1122.42 N for the control (no scaffold). The variance in the response of the nanofiber treatment group was also noticeably less than that of the control (no scaffold) treatment group at the 12-week time point. In fact, if the high and low results for each group were removed from the analysis to reduce the variance range, the nanofiber treatment group demonstrated an ultimate failure force of 1803.55 ± 371.42 N, which was 46.56% higher than that in the control group at 12 weeks. No statistically significant differences in construct stiffness (in newtons per millimeter) were observed within or across time points (*P* = .16; Fig. 3). A statistically significant increase in ultimate failure stress (in megapascals) was observed from the 6- to 12-week time point (*P* < .01), indicating a general increase in failure strength as a function of increased healing time (Fig. 3). No statistically significant differences in construct elastic modulus (in megapascals) were observed within or across time points (*P* = .18; Fig. 3).

### Histopathologic results

**Figure 4:**
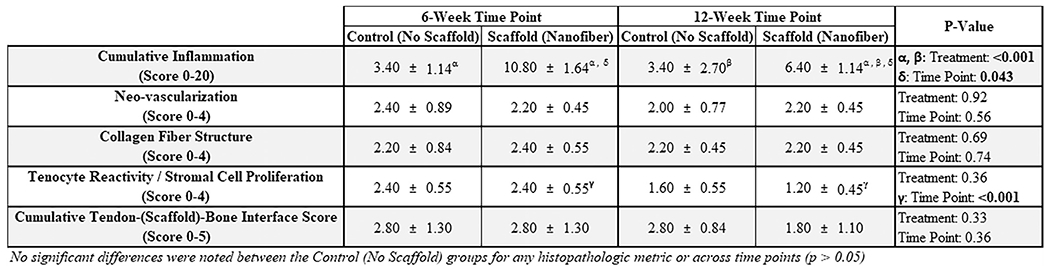
Histopathologic assessment of treatment groups. Like symbols indicate statistically significant differences between groups. The given scores are based on an adaptation of the parameters of the Movin and Bonar scoring systems for specific assessment of tendon histologic characteristics.

Animals in the scaffold-treated group had significantly increased inflammation at the 6-week time point as compared with control animals. This increased inflammatory response was characterized by infiltration of the scaffold material by moderate numbers of macrophages and multi-nucleate giant cells (*P* < .01, unpaired *t* test; Fig. 4). By the 12-week time point, this inflammatory response had subsided and was not statistically significantly different from that observed in control animals (*P* = .052).

**Figure 5:**
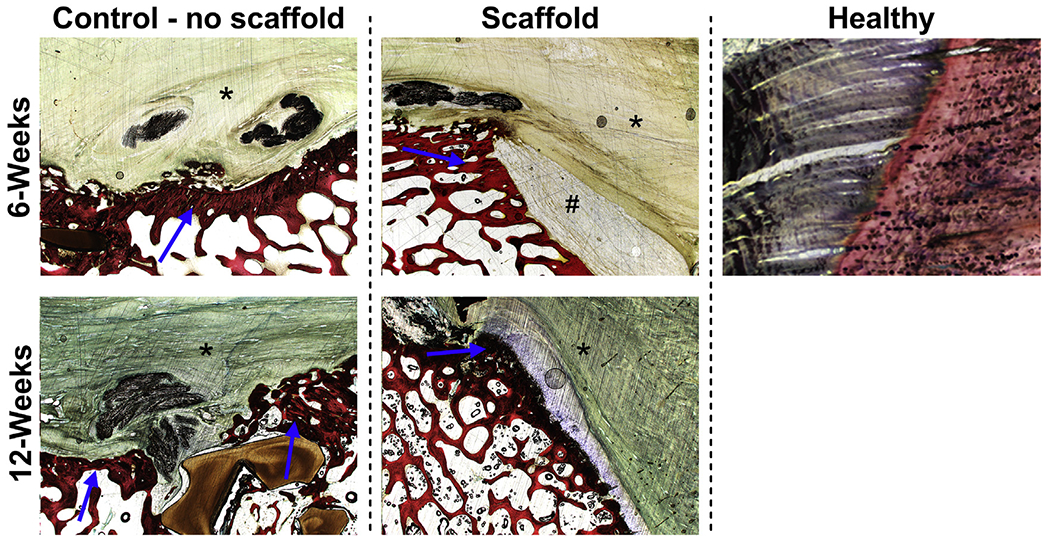
Representative hematoxylin-eosin–stained images (magnification ×1.25) highlighting fibrocartilaginous zone of attachment at tendon-bone interface in both treatment groups at both time points. The enthesis footprint is indicated (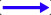), in addition to the tendon (*) and residual scaffold (#) at the 6-week time point.

Qualitatively, histologic evidence of increasing progression of tendon repair from the surgical procedure to the 6-week time point—and then from the 6-week time point to the 12-week time point—in both groups was characterized primarily by (1) re-formation of the tendon-to-bone attachment by 6 weeks after surgery and (2) an increasing transition of the composition of this tendon-to-bone attachment from a primarily fibrous tissue attachment at 6 weeks to a more direct fibrocartilaginous attachment by 12 weeks after surgery. Overall, for both the 6- and 12-week time points, within both the scaffold-treated and no-scaffold groups, there was moderate variation in the character of the tendon-bone interface, as well as the degree to which the normal or native direct-type (fibrocartilaginous) enthesis of the infraspinatus tendon was re-formed. This variation was most pronounced at the earlier, 6-week time point. Overall, for this early time point, no significant histologic differences were observed between animals that received scaffold material and those that did not (Fig. 4). At the 6-week time point, the tendon-bone attachment was most consistent with an “indirect” type of insertion characterized by diffuse fibrous tissue and lesser amounts of cartilage and fibrocartilage broadly attaching along the periosteal surface of the bone. Regarding collagen fiber structure, animals in both treatment groups at this 6-week time point demonstrated increased separation of collagen fibers with loss of parallel arrangement and the presence of newly produced reactive fibroplasia composed of haphazardly arranged collagen bundles. Similarly, the cellularity of both the pre-existing tendon tissue and the newly produced fibrous tissue was increased, characterized by multifocal to diffuse proliferations of tenocytes with rounded plump nuclei and resulting in separation of collagen bundles. In contrast, observations at the 12-week time point displayed a visible difference in the progression and re-formation of the enthesis as compared with those at 6 weeks (Fig. 5).

At the 12-week time point, compared with control animals, animals in the scaffold-treated group displayed an insertion of the fibrous tendon with the humeral footprint that was beginning to be organized in a manner similar to the “native” direct/fibrocartilaginous insertion of the ovine infraspinatus tendon (Fig. 5). This organization of the tendon attachment is reminiscent of the direct/fibrocartilaginous type of enthesis native to the ovine infraspinatus tendons observed in the contralateral tissues. However, it should be noted that in the study animals, the attachment and transition zone of the tendon to fibrocartilage is more diffuse and expanded across the humeral rotator cuff footprint as opposed to a more confined attachment zone typically observed in normal control animals. In addition, and not unsurprisingly for these time points, the organization of the zones of the enthesis (uncalcified and calcified fibrocartilage) is thinner and less prominent than that observed in a normal control animal.

**Figure 6:**
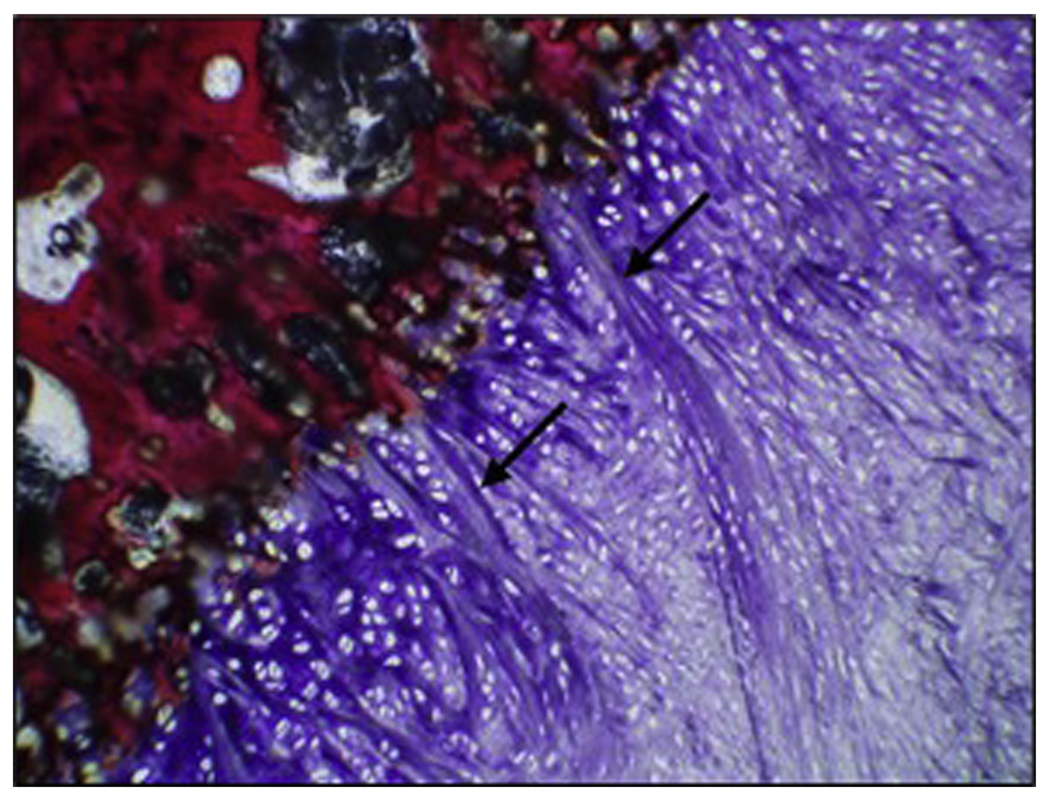
Re-formation of Sharpey-like fibers. A representative image demonstrates the formation of Sharpey-like fibers along a fibrocartilaginous zone of attachment at the tendon-bone interface (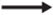). When present, these fibers were characterized as broad, distinctive bundles of dense collagen that originated from the tendon fibrous connective tissue, extended through fibrocartilage or hyaline-like cartilage, and attached to the underlying humeral bone along the tendon-bone interface region of interest (nanofiber treatment, 12 weeks, magnification ×10).

Animals treated with the nanofiber scaffold typically demonstrated prominent perforating collagen fibers, similar to Sharpey fibers, extending through a region of calcified fibrocartilage and attaching to the humeral footprint (Fig. 6). Although this re-formation of a more native direct/fibrocartilaginous enthesis was frequently observed in scaffold-treated animals, this type of direct tendon-bone attachment was not observed between remnant scaffold material (which may take 16 weeks or longer to completely resorb) and the bone surface of the humeral footprint.

## Discussion

Tear and/or degeneration of the tendons in the rotator cuff is one of the most clinically relevant and economically debilitating health issues, affecting up to 70% of Americans and costing >$40 billion in health care annually.^1,16,36,53,54^ Our understanding of conditions that affect tendons is often limited to observing a functional response to a range of treatment modalities without the ability to assess in vivo histologic and biomechanical properties of the tendon. Seventy percent of all shoulder-related symptoms are due to rotator cuff tendon pathology,^46^ which has a detrimental impact on comfort, daily activities, and quality of life.^27^ Most commonly, the ability to compare the effectiveness of various treatments is limited to patient-related outcome measures without knowledge of the histologic or biomechanical effects of treatment. Luckily, the use of a well-developed animal model, such as the infraspinatus tendon in sheep, may provide further insight regarding best treatment practices including the repair of a torn rotator cuff as the biomechanical and histologic properties of the tendon can be assessed in a control untreated group, a control treatment group, and a group with a unique therapeutic intervention.

This study evaluated the biomechanical and histologic effects on rotator cuff tendon healing when using a new, Food and Drug Administration–approved PGA-PLCL nanofiber scaffold between the surgically repaired infraspinatus tendon and its humeral footprint in a sheep model. The surgical technique to implant the graft was easily adapted from current methods for rotator cuff repair and can be performed either in an open manner or arthroscopically in humans.

Most important, this study demonstrated no evidence of an abnormal or pathologic tissue response to the scaffold, along with an improved enthesis architecture and biomechanical properties over the control group. Following the investigation in order of results, biomechanical testing demonstrated statistically significant improvements in ultimate load to failure for both the 6-week group and the 12-week group in the scaffold-treated group. In fact, by 12 weeks, the ultimate load to failure was approaching 75% of the nonsurgical tendon strength, demonstrating not only overall strength benefits but also an accelerated healing response within the scaffold-augmented shoulders. Furthermore, the variance in the results was less with the scaffold than without a scaffold, and all tendons healed despite the fact that the sheep were allowed to re-establish their normal pattern of weight bearing and walking immediately following their recovery from the surgical procedure.

In addition, previous studies have demonstrated ultimate failure loads of 910.4 ± 156.1 N for suture-only controls and 1758 ± 750 N using a poly(lactic-co-glycolic acid) (PLGA) scaffold incorporated into a vented suture anchor.^15^ In our study, with the use of a nanofiber scaffold composed of PGA-PLCL, the ultimate failure load at 12 weeks was 1846.77 ± 431.96 N, approaching the value in the control nonsurgical shoulder, with an ultimate failure load of 2869.96 ± 594.80 N at 12 weeks. The added strength at an earlier time following surgical repair may allow for a more ambitious rehabilitation and strengthening program in addition to preventing the recurrence of a rotator cuff tear, which most commonly occurs in the first 3 months following surgical repair.^30^

It is now accepted that the biological healing cascade is fundamental to achieving a lasting enthesis and, thus, a better clinical outcome.^8^ Furthermore, the typical enthesis that occurs after surgical repair of a torn rotator cuff tendon is not consistent with a restoration of the normal integration of collagen fibers within the bone footprint, yet it may provide a clinically successful functional result. Whereas innovations in mechanical fixation of tendon to bone may facilitate a more consistent biological healing cascade and a sustainable attachment of the tendon to bone, it is remarkable that current healing rates show minimal evidence of improvements over the past 20 years.^14,28^

Most current augmentation devices try to harness the endogenous biological healing following repair^8,13,34,51,52^; however, they have been used primarily as a covering or roof to the mechanical repair technique, away from the actual tendon-bone interface. Mechanically, scaffolds should have sufficient initial strength to withstand the manipulation that occurs with implantation and the physiological loads that occur during the healing process, supported by the fixation method. Biologically, they should support endogenous healing, including the facilitation of cell migration, as well as stability of the microenvironment in terms of growth factors, cells, and vascularity. However, their location away from the actual tendon-bone interface may fail to maximize the ability to augment the biological healing cascade. Structurally, the customized architecture of the scaffolds should provide sufficient pore size and fiber alignment to allow and support cellular migration, persistence of growth factors, vascularization for transporting nutrients and supporting gas exchange, and removal of metabolic end products.^10,24,33,51^ The PGA-PLCL nanofiber scaffold in this investigation is a porous interposition implant that acts similarly to a sponge, attracting and holding on to fluids and their contents. Placing the scaffold between the tendon and bone provides a 3-dimensional architecture that mimics extracellular matrix and attracts and holds on to blood, as well as other fluids, which contain biologically active growth factors and cells from the host. The effect of this interposition graft was not the inhibition of healing but the augmentation seen by the frequent finding of Sharpey-like collagen fibrils penetrating into the bone of the rotator cuff footprint, essentially developing an enthesis that has characteristics of a normal tendon-bone attachment.

Advanced manufacturing technologies have been developed to fabricate this class of scaffolds for rotator cuff repair applications, including electrowriting and electrospinning approaches.^11,13,18,19,31,34,35,44,45,51,55,56,60,61^ Furthermore, the capacity of manufacturing synthetic polymers of various components—or blending with collagen or other materials—allows for an unlimited number of scaffold variations. However, the complexity of the manufacturing, the ability to scale the production of the scaffold, and the ability to achieve cost-effective pricing for these devices will restrict most experimental or investigational scaffolds from widespread human use. Fortunately, polymers such as PGA and PLCL have been shown to be promising base material candidates for such scaffolds as they are readily available, simpler to manufacture, and inexpensive. Electrospinning these base materials results in microporous scaffolds with a high surface area, mimicking extracellular matrix, which is a prerequisite for the possible binding of biologically active molecules and cells.^35^ PGA and PLCL are biodegradable with slow degradation rates in vivo, and PGA- and PLCL-based materials for medical devices have already demonstrated excellent biocompatibility.^12,48,58^ Furthermore, it has been hypothesized that the nonwoven arrangement of the fibers inside these scaffolds, such as the scaffold used for this investigation, may serve as a possible guiding structure for regenerating tendon fibers, in growing blood vessels, and immigrating cells with regenerative capacities, which are the basis for the development of a successfully regenerated tendon-bone transition.^4^

### Limitations

The model used in this study, which was established by Turner,^49^ involves the acute reattachment of a complete tendon laceration, allowing for novel testing of reattachment procedures, fixation devices, and implants that may augment tendon healing. Although this model induces a similar biomechanical profile to human tendinopathy, it creates a more intense inflammatory response than is commonly observed in human disease, although the impact of this response in terms of translation to human tendon healing is not well understood.^7^ This study found the scaffold to be effective in terms of improving biomechanical and histologic findings in the current model without any complications. Translation to human studies is needed to determine the PGA-PLCL nanofiber scaffold’s effectiveness in terms of the tendon healing rate, patient-related outcomes, and quality of life.

## Conclusion

In a sheep acute rotator cuff repair model, securing a nanofiber scaffold between the tendon and the bone using a double-row transosseous-equivalent anchor fixation technique resulted in greater failure strength (1846.77 ± 431.96 N compared with 1247.00 ± 1122.42 N for the group with no scaffold) and a more consistent repair outcome. Additionally, at the enthesis, Sharpey fiber–like attachments (ie, collagen fibers extending from the tendon into the calcified fibrocartilage of the humerus) were observed, which were not seen in the control group. The use of a well-developed animal model, such as the infraspinatus tendon in sheep, may provide further insight regarding best treatment practices as the biomechanical and histologic properties of the tendon can be assessed in a control untreated group, a control treatment group, and an experimental treatment group. The added strength at earlier time points following surgical repair with the Rotium scaffold may allow for a more ambitious rehabilitation and strengthening program in addition to preventing the recurrence of a rotator cuff tear.
